# Commentary: Broca Pars Triangularis Constitutes a “Hub” of the Language-Control Network during Simultaneous Language Translation

**DOI:** 10.3389/fnhum.2018.00022

**Published:** 2018-01-30

**Authors:** Alexis Hervais-Adelman, Barbara Moser-Mercer, Narly Golestani

**Affiliations:** ^1^Neurobiology of Language Department, Max Planck Institute for Psycholinguistics, Nijmegen, Netherlands; ^2^InZone, Geneva Centre for Education and Research in Humanitarian Action, Global Studies Institute, University of Geneva, Geneva, Switzerland; ^3^Brain and Language Lab, Department of Clinical Neurosciences, University of Geneva, Geneva, Switzerland

**Keywords:** simultaneous interpreting, language control, pars triangularis, supplementary motor area (SMA), individual differences

Elmer (2016) conducted an fMRI investigation of “simultaneous language translation” in five participants. The article presents group and individual analyses of German-to-Italian and Italian-to-German translation, confined to a small set of anatomical regions previously reported to be involved in multilingual control. Here we take the opportunity to discuss concerns regarding certain aspects of the study.

A core claim of the article is that group analyses fail to handle individual-differences, especially regarding higher cognitive functions whose loci are putatively more variable across individuals. The utility of using individual participants' functionally-determined regions of interest for analyses has long been considered (Saxe et al., 2006; Fedorenko et al., 2010). However, Elmer does not apply this approach, but rather presents both individual and group-level analyses without formally combining them. A claim is made that this approach is especially beneficial in cases of small sample sizes, but no support exists for this. Even if the approach accommodates variability in the localization of individual participants' activations, the analysis remains an assessment of group-level consistency, and is therefore necessarily subject to the usual concerns regarding statistical power (the problems caused by small sample sizes, including how they have a deleterious impact on the literature by inflating apparent effect-sizes, are discussed in Button et al., 2013). With an estimated effect size of delta = 0.5 (generous for an fMRI contrast), the power to detect a real effect using a one-sample *t*-test at a two-tailed alpha = 0.01 (the uncorrected *p*-value presented in the article) with *N* = 5 is only 3%. The equivalent estimate for the *N* = 50 published by Hervais-Adelman et al. (2015) is 80%.

Crucially for an investigation of simultaneous interpreting (SI), the materials employed do not truly test SI. Short subject-verb-object sentences can be trivially converted between German and Italian as word-for-word calques. This potentially reduces the task to the management of co-activated lexical items, without any requirement to access higher-level linguistic processes (e.g., syntax). Also, participants in this study appear to have initiated their translations, on average, *after* the offset of the sentences with which they were presented (sentences averaged 1.75 s, but mean response latencies reported are > 2.5 s). Seemingly, participants were executing a *consecutive* task rather than a “simultaneous” one. It is therefore questionable whether the reported results relate to SI, when they may in fact relate to the verbal working memory and semantic processes associated with encoding and maintaining the input sentences, rather than language control processes.

Participants in Elmer's study were professional interpreters with expertise ranging from four to 22 years of professional practice. The claim is made that this compensates for the small sample size by estimating a putative impact of expertise, however no analysis of this is presented. Moreover, participants' language combinations are not as well-matched as claimed. If standard definitions of A, B and C languages are used, two of the five participants interpret (consecutively, not simultaneously) into German professionally (those having it as a B language) while the other three do not. This aggravates the issue of individual differences in the Italian-to-German condition.

Elmer's (2016) selection of brain areas for analysis is very restrictive. In contrast, Hervais-Adelman et al. (2015) investigation of SI implicated a broad network of regions, many of which are not considered here, potentially resulting in implicated regions being missed. To enable a more direct comparison, Figure 1 and Table 1 represent analyses analogous to those reported by Elmer (2016), executed on the data from Hervais-Adelman et al. (2015). Namely, we report the proportion of participants showing significant BOLD differences for “Interpreting into L1” vs. “Shadowing L2” at uncorrected *p* < 0.01 in every region of the AAL template (Tzourio-Mazoyer et al., 2002). This analysis shows that the greatest between-subjects consistency in the network (90%) is in left supplementary motor area, a region known to be heavily implicated in cognitive control (Nachev et al., 2008) and language switching (De Baene et al., 2015). Ought we, therefore, conclude that *this* region is the hub of simultaneous interpreting? In the absence of any evidence that can allow us to draw this inference, we would not presume to do so. We therefore question, with such a small sample and without any causal evidence, Elmer's conclusion that the reliability of pars triangularis activation indicates that it is a hub for language control. Elmer's analysis does not consider much of the broad language control network implicated in SI (see Table 1 and Hervais-Adelman et al., 2015), and yet the possibility that regions other than the selected ROIs may be equally or more frequently implicated than pars triangularis is not discussed.

**Figure 1:**
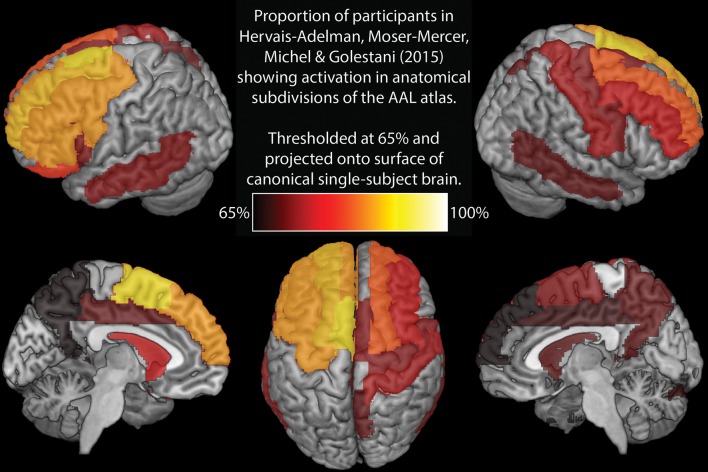
Regions showing significant (at uncorrected *p* < 0.01) activation increase for interpreting vs. shadowing in at least 65% of participants in Hervais-Adelman et al. (2015).

**Table 1:** Proportion of participants in Hervais-Adelman et al. (2015) with significant (at uncorrected *p* < 0.01) activation increase for interpreting vs. shadowing in each region of the AAL template.

**Rank**	**AAL Label**	**%**	**Rank**	**AAL Label**	**%**	**Rank**	**AAL Label**	**%**
1	Supp_Motor_Area_L	90	36	Lingual_R	60	78	Occipital_Inf_R	42
2	Frontal_Sup_L	88	36	Parietal_Sup_R	60	78	SupraMarginal_L^†^	42
3	Precentral_L	86	36	Cerebelum_6_R	60	78	Cerebelum_4_5_L	42
3	Frontal_Mid_L	86	43	Frontal_Sup_Orb_L	58	78	Cerebelum_7b_R	42
3	Frontal_Inf_Tri_L^†^	86	43	Cingulum_Ant_L^†^	58	83	Rolandic_Oper_R	40
3	Frontal_Sup_Medial_L	86	43	Cingulum_Ant_R^†^	58	83	Vermis_6	40
7	Frontal_Sup_R	82	43	Fusiform_L	58	85	Olfactory_L	38
8	Frontal_Inf_Orb_L	80	43	SupraMarginal_R^†^	58	85	Vermis_4_5	38
9	Frontal_Mid_R	76	43	Thalamus_R	58	87	Olfactory_R	36
9	Caudate_L^†^	76	49	Calcarine_L	56	87	Angular_L^†^	36
11	Postcentral_R	74	49	Thalamus_L	56	87	Temporal_Pole_Mid_L	36
12	Precentral_R	72	49	Temporal_Sup_R	56	90	Frontal_Mid_Orb_L	34
12	Frontal_Inf_Oper_L^†^	72	49	Temporal_Pole_Mid_R	56	90	Frontal_Mid_Orb_R	34
12	Supp_Motor_Area_R	72	53	Hippocampus_R	54	90	Rectus_R	34
12	Temporal_Mid_L	72	53	Calcarine_R	54	90	ParaHippocampal_L	34
16	Insula_L^†^	70	53	Occipital_Sup_L	54	90	Angular_R^†^	34
16	Cingulum_Mid_L^†^	70	53	Putamen_R	54	90	Vermis_7	34
16	Precuneus_R	70	53	Cerebelum_6_L	54	96	Rectus_L	32
16	Caudate_R^†^	70	53	Cerebelum_8_R	54	97	Pallidum_L	30
16	Temporal_Mid_R	70	59	Cuneus_R	52	97	Pallidum_R	30
21	Cingulum_Mid_R^†^	68	60	Frontal_Sup_Orb_R	50	99	Heschl_R	28
21	Cerebelum_Crus1_R	68	60	Occipital_Sup_R	50	99	Cerebelum_7b_L	28
23	Frontal_Sup_Medial_R	66	60	Occipital_Mid_R	50	99	Vermis_3	28
23	Fusiform_R	66	60	Paracentral_Lobule_R	50	102	Cingulum_Post_L	24
23	Postcentral_L	66	60	Temporal_Pole_Sup_L	50	102	Cerebelum_3_L	24
23	Precuneus_L	66	65	Parietal_Inf_R^†^	48	102	Cerebelum_10_R	24
27	Lingual_L	64	65	Putamen_L	48	105	Cingulum_Post_R	22
27	Parietal_Sup_L	64	65	Cerebelum_Crus2_L	48	105	Heschl_L	22
27	Temporal_Inf_L	64	65	Cerebelum_4_5_R	48	105	Cerebelum_3_R	22
27	Temporal_Inf_R	64	69	Frontal_Mid_Orb_R	46	105	Cerebelum_9_R	22
31	Insula_R^†^	62	69	Hippocampus_L	46	109	Amygdala_R	20
31	Occipital_Mid_L	62	69	Cuneus_L	46	109	Cerebelum_10_L	20
31	Parietal_Inf_L	62	72	Rolandic_Oper_L	44	111	Vermis_8	16
31	Cerebelum_Crus1_L	62	72	ParaHippocampal_R	44	112	Amygdala_L	12
31	Cerebelum_Crus2_R	62	72	Paracentral_Lobule_L	44	112	Cerebelum_9_L	12
36	Frontal_Mid_Orb_L	60	72	Temporal_Sup_L	44	112	Vermis_1_2	12
36	Frontal_Inf_Oper_R^†^	60	72	Temporal_Pole_Sup_R	44	115	Vermis_9	10
36	Frontal_Inf_Tri_R^†^	60	72	Cerebelum_8_L	44	116	Vermis_10	6
36	Frontal_Inf_Orb_R	60	78	Occipital_Inf_L	42			

† *denotes those regions that were considered by Elmer (2016): pars triangularis, pars opercularis, middle and anterior cingulate, caudate nuclei, supramarginal gyrus, angular gyrus and anterior insulae. We note with interest that the ROIs included in Elmer's study only include two of the ten most consistent regions found in these data*.

We do not question that pars triangularis plays a substantial role in interpreting, but the data do not provide emphatic support for the idea that “These results challenge previous models” nor do they suggest the need for “re-definition of the language-control network” (Elmer, 2016, p.5). Although we appreciate that the paper incorporates an extensive “limitations” section, those limitations are seemingly not taken into consideration when drawing these conclusions. The paper contains some genuine issues beyond those acknowledged that we worry fundamentally undermine the conclusions: real effects are likely to have been missed due to lack of power, the participant selection introduced unnecessary sources of variability (age and expertise), the selection of materials means that the reported effects may not relate to SI but to consecutive interpretation and the constrained analysis space rules out conclusions about the broader language control network. These, coupled with the statistically-questionable claims made regarding how the small sample size and inter-subject variability can somehow be overcome, lead us to fundamentally question the conclusions of the article.

We welcome all challenges that arise from any effort to replicate and improve upon our and others' studies. However, while cognitive neuroscience finds itself in the harsh spotlight of a “reproducibility crisis” (Barch and Yarkoni, 2013), it behooves us to be cautious in our approach to publication, and it seems especially important to avoid drawing overly strong conclusions on the basis of underpowered studies.

## Author contributions

AH-A, BM-M, and NG wrote the manuscript. AH-A carried out reanalyses.

### Conflict of interest statement

The authors declare that the research was conducted in the absence of any commercial or financial relationships that could be construed as a potential conflict of interest.
